# Wildfire Detection Using Sound Spectrum Analysis Based on the Internet of Things

**DOI:** 10.3390/s19235093

**Published:** 2019-11-21

**Authors:** Shuo Zhang, Demin Gao, Haifeng Lin, Quan Sun

**Affiliations:** College of Information Science and Technology, Nanjing Forestry University, Nanjing 210037, China; zhangshuo@njfu.edu.cn (S.Z.); haifeng.lin@njfu.edu.cn (H.L.); sunquan@njfu.edu.cn (Q.S.)

**Keywords:** wildfire, Internet of Things, sound spectrum analysis, tree-energy device, crown fire, surface fire

## Abstract

Wildfire is a sudden and hazardous natural disaster. Currently, many schemes based on optical spectrum analysis have been proposed to detect wildfire, but obstacles in forest areas can decrease the efficiency of spectral monitoring, resulting in a wildfire detection system not being able to monitor the occurrence of wildfire promptly. In this paper, we propose a novel wildfire detection system using sound spectrum analysis based on the Internet of Things (IoT), which utilizes a wireless acoustic detection system to probe wildfire and distinguish the difference in the sound between the crown and the surface fire. We also designed a new power supply unit: tree-energy device, which utilizes the biological energy of the living trees to generate electricity. We implemented sound spectrum analysis on the data collected by sound sensors and then combined our classification algorithms. The results describe that the sound frequency of the crown fire is about 0–400 Hz, while the sound frequency of the surface fire ranges from 0 to 15,000 Hz. However, the accuracy of the classification method is affected by some factors, such as the distribution of sensors, the loss of energy in sound transmission, and the delay of data transmission. In the simulation experiments, the recognition rate of the method can reach about 70%.

## 1. Introduction

Wildfire destroys millions of hectares of forest, pollutes the environment, causes severe casualties, and has a significant economic impact on government budgets every year [1,2]. Detecting a wildfire promptly, before it is out of control, is still a difficult challenge. According to the combustion materials, a wildfire is generally classified into three types: underground fire, surface fire, or crown fire. An underground fire is caused by spontaneous combustion or combustion in other channels after coal strata meet combustion conditions under the surface; and a surface fire, when not acted upon by external forces, usually spreads along the surface of forest areas. The surface fire is easily affected by wind, causing the flames to disperse everywhere, eventually falling on the crown and branches, causing a crown fire to occur. Crown fires have exceptionally high temperatures and ferocious behavior, which makes them challenging to be extinguished, making them extremely dangerous [3]. In addition, a crown fire always spreads more than 100 times faster than a surface fire and is more destructive. Thus, because of this phenomenon, it is essential to determine the type of wildfire as early as possible in early wildfire detection, for the sake of adopting efficient strategies to fight the wildfire and reduce the casualties and economic losses.

Nowadays, due to the rapid development of imaging techniques and the full use of the spectral camera, contemporaneously, optical spectrum analysis technology [4] is widely used for perceiving the ignition of wild land, such as wildfire video monitoring [5], remote sensing [6], etc. The working principle of these technologies is photogrammetry at visible and infrared bands on the forest surface, and automatic detection of fire evidence by fixed field cameras mounted on control towers or by radar, or by mobile cameras mounted on any aircraft (aircraft, unmanned aerial vehicles (UAVs), air balloons, or satellites). However, there are many obstacles in forested areas, and the optical spectrum analysis technology is vulnerable to external interference. Thus, wildfires cannot always be detected promptly. Additionally, the speed of wildfire spreading is high, especially in the face of a strong wind, which leads the wildfire being out of controlled and causes casualties [7,8]. In addition, wild regions are generally far away from infrastructure, so it is impractical to render a video device due to the energy requirement. Therefore, we still face challenges in early wildfire detection.

It is common that during the progress of wildfire burning, it generates noise. Based on this phenomenon, we propose a new research direction; that is, using sound spectrum analysis for the early wildfire detection. It was found that one of the most efficient approaches to finding out the information of a signal at varying frequencies is spectrum analysis. Spectrum analysis technology can decompose complex noise signals into relatively simple signals [9]. Therefore, drawing support from this technology, many physical signals can be represented by the sum of many simple signals of various frequencies [10,11]. Spectrum analysis can process the whole signal, but it sometimes divides the signal into several segments [12,13]. Moreover, a sound source can also be made up of many varying frequencies [14]; according to related work, different frequencies stimulate the corresponding parts in the ear [15]. Compared to optical spectrum analysis, the use of sound spectrum analysis is more effective and timely to detect wildfires, because the sound transmission in forest areas is less susceptible to external interference than the light transmission. Hence, the propose of this work was to detect wildfires in time and find a simple criterion for instantaneous differentiation between crown and surface fires to be utilized in the Internet of Things (IoT) for wildfire early detection.

When there is no wildfire in the forestry area, most of the sounds produced in the forests come from animals’ activities, which is an irresistible external influence. But unlike the noise of wildfire, the sound of animals is not continuous [16]. Therefore, when we analyze the sound spectrum of the data collected by the sound sensor, we can easily distinguish the sound of wildfire from the sound of animals. However, forests generally occupy a large area and a rugged terrain, requiring quantitative sensors to be carefully deployed. Besides, we also need to consider the energy loss of sound during propagation and the possibility of data distortion and delay in the use of wireless sensor networks for data transmission, all of which will reduce the accuracy of our method. After measuring, the accuracy of our classification method can reach about 70%.

In our work, we attempted to implement a wildfire detection system using the sound spectrum analysis based on the Internet of things which is powered by the tree-energy device we designed. This device utilizes the bio-energy of the living trees to generate electrical energy. To further communication distance and decrease the energy consumption of the sensors, we choose an LoRa device that can consume less energy and transmit over long distances for data transmission. The method we propose not only solves the problem of the limited power of the sensors’ batteries but also deals with the short communication distance of the sensor, which makes our early wildfire detection model better suited for continuous wildfire monitoring.

The main work can be summarized as follows:

- We designed a tree-energy harvesting device relying on a constant potential (voltage) difference between the xylem of the plant and the surrounding soil, which can provide stable power for our monitoring devices and make our network operate normally and perpetually.
- We designed a sound collection system based on Internet of Things technology to collect wildfire sound and used spectrum analysis technology to analyze the ignition of wild land.
- Based on the spectrum analysis technology, crown fires and surface fires can be recognized in our model. From the experiment, results illustrate that our design is effective for detecting wildfire.

The rest of this paper is organized as follows. In Section 2, we give a review on the existing wildfire monitoring technologies. Section 3 explains our system model. In Section 4, we discuss the practical experiment results. Section 5 concludes the paper with a brief summary.

## 2. Related Work

Heretofore, various wildfire monitoring technologies have been proposed and employed to aim at detecting initial wildfire. These early detection technologies are divided into several categories: ground patrol, remote video monitoring [17], artificial tower observation [18], forest aviation patrol [19], satellite remote sensing [20], wireless sensor networks [21], IoT [22], etc. The following are the advantages and disadvantages of these technologies.

The artificial observation of the tower is built by making use of the characteristics of the open view and the high terrain of the mountain to observe and locate the fire field through simple devices for fire monitoring and fire warning. It has an excellent and visualized monitoring effect, but the setting and monitoring effect is restricted by the objective conditions, such as geography and weather. As a result, it is hard to observe large-scale forests comprehensively and discover the occurrence of fires timely. Additionally, some objective factors such as large patrol area, narrow field of view, and low efficiency, which are often affected by the complexity of the topography, make it difficult to determine the location of a fire’s source.

Forest aviation patrol is a means of real-time forest fire detection and cruise protection for forests by using helicopters or drones [23,24]. It has a wide field of vision, high speed, and high mobility, and can comprehensively watch and monitor the surrounding environment and the spread of a fire. However, this monitoring technology is greatly affected by climate and local weather, and has a high construction and operation cost [25].

Satellite remote sensing via in-orbit satellite real-time monitoring, with the aid of space remote sensing technology, can probe the hot spot location of wildfire and analyze the information of fire spread [26,27]. It has high continuity and is one of the most advanced monitoring means for fire protection technology. Nevertheless, it is usually affected by some elements, such as cloud thickness and water content over the fire scene; consequently, this technology is regularly used as an early warning method [28]. Besides, satellite data have a low spatial resolution, and the wildfire monitoring using thermal infrared channel data is easily interfered with by a sturdy reflection surface, high-temperature saturation, and other factors, which is not conducive to full tracking of a wildfire’s intensity spatial pattern [29].

Compared with the above monitoring technologies, wireless sensor networks (WSNs) are some of the most reliable tools for the early detection of wildfires. WSNs are made up of tiny, cheap, and low-power sensor devices that can measure environmental traits [30]. This method generally has the ability to measure and monitor environmental parameters (temperature, relative humidity, carbon monoxide, carbon dioxide, rainfall, etc.) using sensors located in the controlled forest territory, together with data transfer to the tracking center through a network in real time. If some of these measured parameters are above the configured thresholds, the system analyzes the information and reacts, sending an alarm to the fire fighters or the operators devoted to forest fire monitoring. The fire sites can be located instantly with accuracy because each sensor can integrate a GPS receiver. WSNs can quickly transmit the data collected in the wildfire site to the base station through wireless communication [31]. Unfortunately, the traditional wireless sensor networks cannot have an unlimited life without battery charging or replacement. All sensor nodes need battery power, and the power supply capacity directly affects the energy and data processing capacity of nodes [32].

According to the architecture of the Internet of things and the characteristics of wildfire monitoring systems, wildfire monitoring based on the architecture of the Internet of things consists of three parts: a wireless sensor network responsible for data collection, a wireless sensor network and existing network for remote data transmission, and a wildfire monitoring center [33]. The wireless sensor network collects data for the wildfire monitoring system, which can detect the parameters of each monitoring node in the target area. Then, it will transmit the collected data to the aggregation node through the intermediate routing node [34]. After that, the aggregation node sends data to the wildfire monitoring center through the existing wired or wireless communication network [35]. The wildfire monitoring center analyzes and processes the critical factors of wildfire through the transmitted monitoring data, providing auxiliary decision-making for wildfire monitoring.

The Internet of Things can be applied to monitor in many essential prevention areas. According to the needs of wildfire factor monitoring in wild land, it can establish an information collection, analysis, and early warning system based on wireless sensor networks through the wireless sensor network’s ability to accurately detect complex environments and emergencies. Besides, it can monitor the dynamics of forests more timely and effectively, and reduce the destruction of terrestrial ecosystems and air pollution caused by fires.

The function of the sound spectrum analysis technique is to obtain more accurate spectral characteristics by some operations, such as Fourier transform [36]. As a modern signal analysis method, spectrum analysis has been widely adopted in various disciplines as an essential basis for the research, production, and inspection of different electronic products. Because of its implementation of high-resolution, wide-band digital spectrum analysis, it has been the focus of research in this field, such as speech recognition in industrial control, telecommunications systems, etc. Nowadays, in the field of forest noise monitoring, sound spectrum analysis technology is utilized to prevent illegal logging and monitor animal activity [37]. According to the comparison of animal sound spectrum database, animal activity in the forest can be identified [38]. In the literature [39], the authors analyze and classify the sounds produced by some amphibian species. Before the spectrum analysis, the sound source signal needs to be weighed by frequency. The frequency weighting filter is mainly implemented through an analog circuit, and the amplified sound pressure signal passes through a frequency weighting filter circuit to obtain the time domain signal after weighing, which is composed of capacitance, resistance, inductance, etc. With the increasingly mature application of Fourier transform technology and the rapid development of computer software technology, this technology is increasingly sophisticated.

Different from traditional wildfire monitoring systems based on optical spectrum analysis, we introduce a new wildfire detection system utilizing sound spectrum analysis based on IoT. Compared with optical spectrum analysis, the advantage of using sound spectrum analysis to detect wildfires is that the influence of obstacles in the forest on the sound data collected by sensor nodes can be ignored, which helps fire departments to monitor wildfires promptly. Besides, this design takes advantages of the technology: converting tree energy into electrical energy to provide power for the sensor nodes, which successfully replaces the traditional, disposable chemical battery and effectively solves the problem of insufficient energy supply.

## 3. System Design

**Figure 1:**
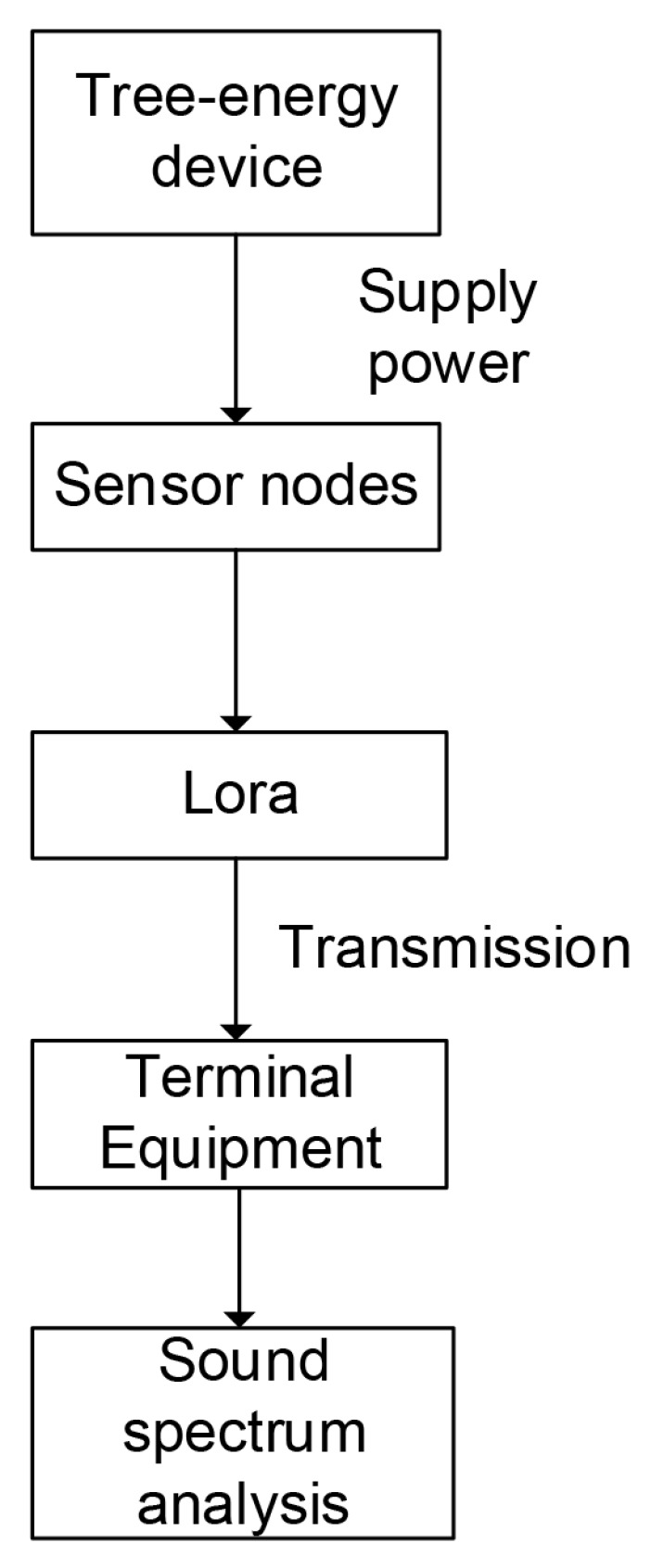
An overall flowchart of system.

In this section, we present a deep description regarding our wildfire detection system which employs the sound spectrum analysis method based on the Internet of Things. An overall flowchart of the proposed design is shown in Figure 1. This section is divided into four parts (the principle of energy harvesting from living trees, tree-energy device design, sound collection and transmission, and sound spectrum analysis) to describe our wildfire detection system specifically.

### 3.1. The Principle of Energy Harvesting from Living Trees

In the previous section, we discussed the energy supply issue of existing wildfire detection technologies, while the traditional solution is to use disposable chemical batteries with limited energy to provide power for conventional wireless sensor networks [40]. The reason why batteries are chosen to power nodes is that forested areas are generally in remote areas, and the terrain is rugged, which is unfavorable for supplying power to the sensors and to the data transmission of the sensors. It is universally acknowledged that it is inconvenient to replace disposable chemical batteries with limited energy in forested areas, and these batteries will cause severe pollution to the environment. To solve this issue, we did some related studies and found that we could harvest energy from living trees.

#### 3.1.1. The Voltage Caused by Differences in the pH of the Xylem and Soil of Trees

**Figure 2:**
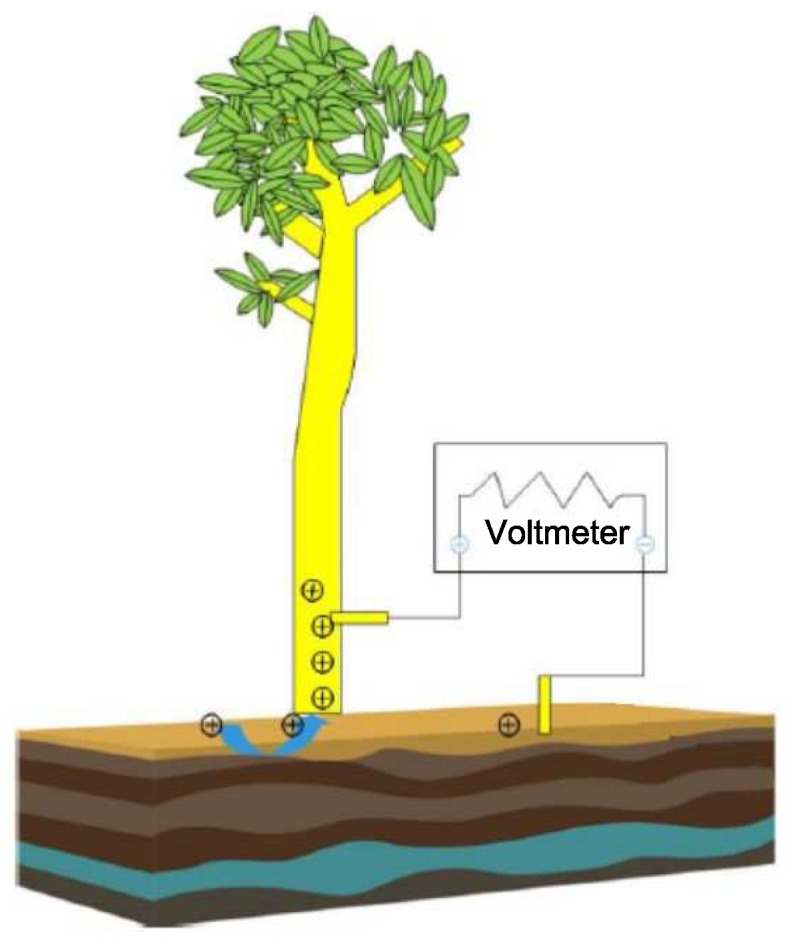
Schematic diagram of tree-energy acquisition.

In the process of measuring the voltage of eucalyptus, the Nernst equation generated from the potential voltage of the pH concentration is expressed as: $V=V^{\prime }-\frac{RT}{nF}\left[\Delta pH\right]∼56mV\left[\Delta pH\right]$, where R is a general gas constant of 8.314 JK^−1^ mol^−1^, T is the Kelvin temperature, and F is the electric charge times Avogadro’s constant, which is 9.648 $\times 10^{4}$ C mol^−1^, and [ΔpH] is the difference between the two pH values [41]. This formula can be used to calculate the theoretical power that Eucalyptus can produce. From Figure 2, we can observe that under the effect of the tree’s metabolism, it can produce a weak current.

#### 3.1.2. The Voltage Generated by Fluid Flow in the Xylem of a Tree

We inserted two identical platinum electrodes into the wooden part of the tree (removing the phloem) and connected the electrodes to a high-impedance voltmeter. The mechanism of continuous potential voltage generation depends on the electric potential voltage difference (ξ). Due to the action of the cell wall, the liquid in the central capillary has flow characteristics and a pressure difference $V_{sapstream}=\frac{\varepsilon_{0}\varepsilon_{r}}{\sigma \eta }\Delta P\xi$ is formed at both ends, where $\varepsilon_{0}$ represents the permeability of the vacuum medium, $\varepsilon_{0}$ = 8.85 $\times 10^{-12}$ F/m (C^2^/Jm), $\varepsilon_{r}$ indicates the dielectric constant of the xylem, $\varepsilon_{r}=$ ∼80, σ is the conductivity, σ= ∼0.01 S/m, η is the viscosity, and η= ∼10^−3^ Pas. Δp represents the pressure difference, ξ represents the electric potential, due to the difference in fluidity between the liquid and the pore wall atoms, Δp= ∼1 MPa, ξ=0.1 V, according to the previous experimental measurement, $V_{sapstream}=$ ∼(1–10 mV), the voltage value obtained from the tree is between 1 and 10 mV [41]. According to the above formula, the faster the flow rate of the liquid-liquid flow in the tree, the higher the voltage generated, and different tree types will produce different voltage differentials.

### 3.2. Tree-Energy Device Design

Nowadays, a wireless sensor network is the most time-effective tool applied in the field of wildfire detection, but the power required for its working comes from the batteries with limited energy, which shorten its service life and brings enormous operational pressure to wildfire departments. With these issues, it will cause some adverse consequences; for instance, the wildfire department cannot discover the fire in time if the communication is interrupted. At present, the batteries of wireless sensors are rechargeable, and most of them can get recharged using solar energy. Collecting energy from the sun is a promising technology that can continuously drive wireless sensor networks, but tall trees and other barriers will obscure the solar panels, and cells’ charging will be affected easily by rainy weather. As a result, the batteries sometimes fail to recharge in time, resulting in insufficient power for the nodes.

In the works [41,42], the authors notice that the voltage difference between the wooden parts of the trees and the soil, in which they grow. This discovery offered another opportunity to provide power for wireless sensor networks and helped to solve the problem of energy supply. On account of this finding, we designed the tree-energy device that can extract energy from living trees. We employed this device as a battery in a wireless sensor to monitor the environment and data transmission based on the IoT.

In accordance with the characteristics of plants, there is a constant potential (voltage) difference between the wooden parts of the plants and the surrounding soil. In the experiments, we measured the pH values between soil and the wooden parts of living trees. The results show that there is 50–200 mV of continuous voltage difference between the xylem and the soil. Through repeated experimental measurements, we know that there is no correlation between voltage and external factors such as time, light, juice flow, electrode height, or the ionic composition of soil, indicating that a metal redox reaction cannot cause voltage difference. Meanwhile, the pH value between the xylem and the soil can affect the polarity and amplitude of the voltage. These sustained voltages are originated from the concentrated bio cells established by the steady-state mechanism of the trees. To measure the voltage between the wooden parts and the soils with different pH values, we took some action, including using RF noise pickup, current, and different metal redox reactions on the electrode to control the voltage source.

**Figure 3:**
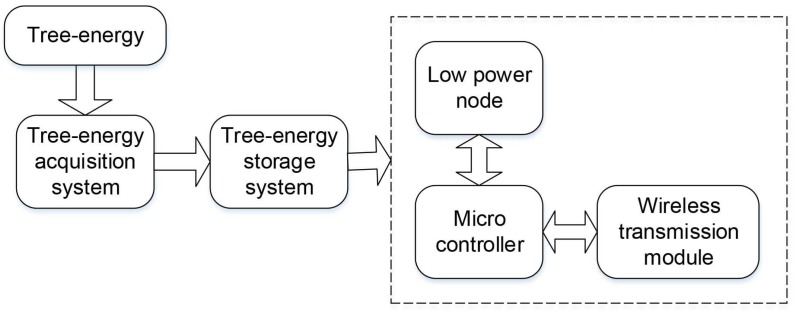
Wireless sensor powered by tree.

In terms of the functional structure of the circuit, the wireless sensor powered by tree-energy device is subdivided into two parts: the energy and communication module. Among them, the energy module undertakes the tasks to convert the tree-energy into electrical energy and store the electrical energy in its storage, while the components of communication module mainly contain low-power nodes, microcontrollers, and wireless transmission, as shown in Figure 3.

Relying on the related work [42] that the voltage difference between the wooden parts of the trees and the soil in the planted area, we designed the tree-energy generated device. In the design, we use a multi-stage capacitor and multi-switch control power transmission to harvest tree-energy. At the same time, this paper also introduces the design and implementation of transformers and DC/DC boost converters. It not only improves the weak voltage borne by tree-energy but also guarantees the stability of voltage in the process of power supply. The purpose of obtaining a more stable and efficient power output for the power supply is to achieve a variety of electrical applications.

Living trees can be thought of as a power source and linked to capacitor C_1_. In the initial state, a certain amount of low-voltage current exists in the capacitor C_1_, which is grounded to protect electrical components. Besides, the capacitor C_1_ needs to be connected to S_1_ that can be used to prevent the transformer and DC/DC booster converters from drawing the current out of capacitor C_1_ while charging. Once the voltage of capacitor C_1_ reaches the voltage required by the hardware, S_1_ is closed and C_1_ serves as the power source to drive the rest of the energy transfer circuit, as well as the nodes.

The transformer is connected to the S_1_ and DC/DC converter, which is used to amplify the voltage of the capacitor C_1_. The DC/DC converter converts the energy at the input of the transformer and then effectively outputs a fixed voltage. When the transformer is discharged, the capacitor C_2_ begins to be charged. Once the voltage of C_1_ drops below the charging voltage, S_1_ will open and C_1_ will start being charged again. This process is iterated several times until the voltage at C_2_ reaches the desired node voltage. When S_2_ is closed, power for nodes is supplied by C_2_. The function of switch 2 is to connect the node when C_2_ is fully charged, and prevents the load and current from flowing backward during C_2_ charging.

**Figure 4:**
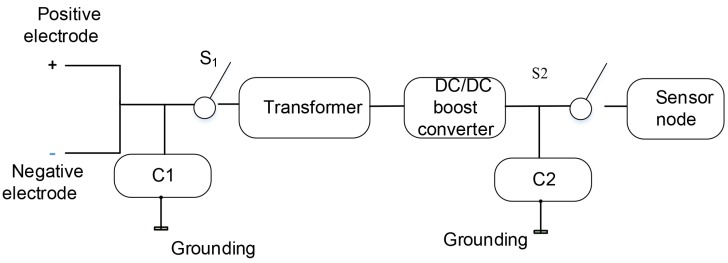
Schematic diagram of the tree-energy generation device.

The capacitor of C_1_ is set to 0.22 F, and the charging voltage is 350 mV, while the discharging voltage is 100 mV. The capacitor of C_2_ is set at 2.5 F. When C_1_ is charged to 350 mV, S_1_ closes and starts charging C_2_. After C_2_ being completed charged, S_2_ closes and begins to drive the wireless sensor node. When the voltage of C_2_ drops to 2.4 V, S_2_ is turned on and C_2_ starts to be recharged. The working/sleeping mode switch of the wireless sensor node can be adjusted by S_2_. Since the power consumption in the sleeping mode is close to zero, C_2_ can be charged continuously, as shown in Figure 4.

Table 1 is the abbreviation of the name of the experimentally related components.

### 3.3. Sound Collection and Transmission

**Figure 5:**
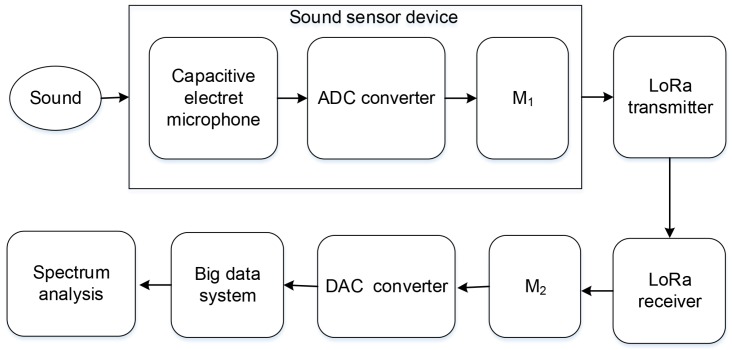
The architecture of sound spectrum analysis.

To adjust the energy expenditure, the sensor node adjusts its duty cycle based on available energy or application requirements. In the case of bursts and high traffic loads, the duty cycle of the nodes can be increased to meet requirements, such as low latency and high reliability. That is, nodes will wake up more frequently to reduce end-to-end latency. However, high duty cycles can result in significant energy consumption. Therefore, we set the duty cycle to be about 10%, which means that 10 min is a working cycle. In 10 min, the nodes only work for one minute, and the rest of the time is in a dormant state for energy that can be saved to the utmost. In the design, we employed the sound sensor to collect sound signals. The Figure 5 plots the architecture of sound spectrum analysis.

The electret microphone is installed inside the sound sensor, and it mainly consists of two parts—the acoustic-electric conversion part and impedance part. Sound waves vibrate the electret membrane inside the microphone, causing capacitance to change and producing a small voltage corresponding to the change. Then, the voltage is converted to 0–5 V and the sound signal to the electrical signal. Instantly, the ADC (analog-to-digital converter) receives the electrical signal and converts the analog signal of the sound source into a digital signal. After the M_1_ reads the measured value of the sensor, the conversion is carried out, and the data is transferred to the register at the transmitter of LoRa. When data processing is completed, M_1_ sends a single-step command to the sensor, and the single-step command causes the sensor to initiate a sound detection and then automatically enters a standby mode. Subsequently, the digital signal is sent to the receiver through the LoRa transmitter. The next step is that M_2_ reads the value measured received by the receiver, converts the binary code to BCD (binary-coded decimal) code, and transfers the data to the register. Then, the DAC (digital-to-analog converter) converts the digital signal into an analog signal for transmission to the PC. Finally, MATLAB is utilized to analyze the sound frequency of the collected sound.

In this paper, LoRa technology is adopted to achieve long-distance communication. As a gateway mode, LoRa can connect a certain number of wireless sensor nodes and replace the traditional GPRS (general packet radio service) module, effectively reducing the cost. If LoRa is used as the relay node, the data can reach hundreds of kilometers in the multi-hop mode.

**Figure 6:**
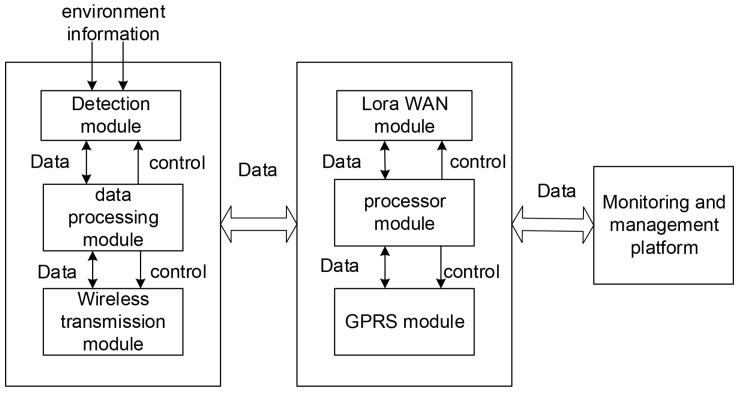
The architecture of remote monitoring system.

The architecture of remote monitoring system based on LoRa is shown in Figure 6. In the data acquisition subsystem for various applications, there are a large number of sensors embedded with RF module. The information collected by them is sent to the sub-database of the corresponding application through one or more gateways and displayed in the monitoring and management platform. Then users can log in the monitoring management platform as customers or administrators for data query, node management, etc.

LoRa devices can communicate in point-to-point mode with low power consumption and low data transmission rate. Under barrier-free conditions, communication distance between LoRa devices can reach 15 km. The reason why the LoRa module can be quickly applied to various industries is that it has certain features, such as cabling-free and anti-interference capability. However, in practical applications, we are unable to achieve barrier-free transmission, because obstacles such as trees, hillsides, and rivers in the forests will affect the transmission distance of the LoRa module. In a forested area, we measured that the range of LoRa module data transmission can only reach about 3 km. Therefore, for avoiding affecting project data uploading, users should depend on specific field conditions to choose to employ the LoRa module.

### 3.4. Sound Spectrum Analysis for Wildfire

Different combustion materials produce different noise frequencies during the progress of burning. As reported in the scientific literature, the frequency range of crown fire is relatively narrow, and we expect the sound spectrum of crown fires to range from 200 to 450 Hz. The existing method to define the wildfire type by noise is to compare the measured wildfire noise with the known wildfire noise spectrum in the database. However, this method lacks timeliness and accuracy and is not suitable to be applied for sensor microprocessors. The purpose of this study was to find a simple division standard for the crown and surface fire for early wildfire detection using sound spectrum analysis based on the Internet of things.

We utilized Fourier transform to analyze the noise spectrum characteristics of different wildfire, and propose a wildfire detection method based on sound spectrum analysis. The purpose of this method is to identify different features of the noise frequency response of wildfire types. The records of wildfire noise were taken from the data collected by sensors deployed in forests.

The sampling theorem is vital in digital signal processing. It describes the relationship between the signal frequency and the sampling frequency to be satisfied when the analog signal is converted into a digital signal. Therefore, we obtain the discrete data by the sampling theorem. The analog signal $x_{a}\left(t\right)$ is ideally sampled at equal intervals in time domain with interval T, and the spectrum of the sampled signal formed $\hat{X}\left(j\Omega \right)$ is the original analog signal spectrum $X_{a}\left(j\Omega \right)$. The cycle is extended with a sampling angle frequency $\Omega_{s}\left(\Omega_{s}=\frac{2\pi }{T}\right)$. The formula is:

(1) $$\begin{array}{c} {\hat{X}}_{a}\left(j\Omega \right)=FT\left[{\hat{x}}_{a}\left(t\right)\right]=\frac{1}{T}\sum_{n=-\infty }^{+\infty }x_{a}\left(j\Omega -jn\Omega_{s}\right), \end{array}$$

where the sampling frequency $\Omega_{s}$ must be greater than or equal to more than twice the highest frequency of the analog signal $x_{a}\left(t\right)$ for the spectrum of the sampled signal to not be spectrally aliased.

First, we assume that the relationship between the ideal sampled signal ${\hat{x}}_{a}\left(t\right)$ and the analog signal $x_{a}\left(t\right)$ is:

(2) $$\begin{array}{c} {\hat{x}}_{a}\left(t\right)=x_{a}\left(t\right)\sum_{n=-\infty }^{+\infty }\delta \left(t-nT\right). \end{array}$$

Performing Fourier transform on Equation (2), we get:

(3) $$\begin{array}{c} {\hat{X}}_{a}\left(j\Omega \right)=\int_{-\infty }^{+\infty }\left[x_{a}\left(t\right)\sum_{n=-\infty }^{+\infty }\delta \left(t-nT\right)\right]e^{-j\Omega t}dt=\sum_{n=-\infty }^{+\infty }\int_{-\infty }^{+\infty }\left[x_{a}\left(t\right)\delta \left(t-nT\right)\right]e^{-j\Omega t}dt \end{array}$$

In Equation (3), there is a non-zero value only when t=nT in the integral number, then we rewrite Equation (3) as:

(4) $$\begin{array}{c} {\hat{X}}_{a}\left(j\Omega \right)=\sum_{n=-\infty }^{+\infty }x_{a}\left(nT\right)e^{-j\Omega nT}. \end{array}$$

Let $x_{a}\left(nT\right)=x\left(n\right)$, ω=ΩT and substitute it into Equation (4):

(5) $$\begin{array}{c} {\hat{X}}_{a}\left(j\Omega \right)=\sum_{n=-\infty }^{+\infty }x\left(n\right)e^{-j\omega n} \end{array}$$

Obviously, the right side of Equation (5) is the Fourier transform of the sequence $X(e^{j\omega })$, which is:

(6) $$\begin{array}{c} {\hat{X}}_{a}\left(j\Omega \right)=X\left(e^{j\omega }\right){|}_{\omega =\Omega T}. \end{array}$$

Equation (6) shows that the Fourier transform of the ideal sampled signal can be obtained by Fourier transform of the corresponding sample sequence.

We assume that ${\hat{X}}_{a}\left(j\Omega \right)=X\left(n\right)$, and *n* denotes the fire combustion noise obtained instantly, corresponding to the serial number of the analog signal. To determine whether the noise of wildfire corresponding to the analog signal $x_{i}$ is a crown fire, we define the spectrum of signal $C_{n}$, which is obtained by adopting cross-spectrum. For the two stationary random signals $X_{n}$ and $S_{n}$, according to the stochastic process theory, the statistical correlation between them should be expressed by their cross-correlation function. The Fourier transform is performed on the cross-correlation function of $X_{n}$ and $S_{n}$ to obtain the power density spectrum in the frequency domain, which is called the mutual power density spectrum. More specifically,

(7) $$\begin{array}{c} C_{n}=\sum_{\alpha =1}^{n}S_{\alpha }X_{n}, \end{array}$$

where we suppose that the range of α is from 1 to *n*, and $S_{1}$, $S_{2}$, ⋯, $S_{\alpha }$, ⋯, $S_{n}$ represent the frequency values of the analog signal, corresponding to the noise wildfire obtained by the sound sensors. Depending on Equation (7), we can attain the signal spectrum $C_{n}$ corresponding to the analog signal $x_{i}$.

Cross-spectrum is an abbreviation for cross-power density spectrum, a method of describing the degree of statistical correlation between two different signals in the frequency domain. Then, we perform a polynomial fit on the trend line corresponding to the signal spectrum $C_{n}$ to obtain the trend line slope coefficient $k_{a}$. The power spectrum of the fire combustion noise, $P_{f}$, can be written as:

(8) $$\begin{array}{c} P_{f}=\sum_{a=0}^{\beta }k_{a}{\left(f_{a}\right)}^{a}. \end{array}$$

In Equation (8), we assume that 0≤a≤β, while β represents the maximum polynomial number in the polynomial corresponding to each segment of the trend line. $k_{a}$ represents the slope coefficient of the segment of the *a* degree polynomial on the trend line, and $f_{a}$ denotes the frequency value of the section of the *a* degree polynomial on the trend line and the simulated signal of fire combustion noise.

According to the trend line corresponding to the signal spectrum $C_{n}$, the sum of squares of the low frequency spectrum amplitude and the high frequency spectrum amplitude of each analog signal $x_{n}$ is calculated for the analog signal $x_{n}$ corresponding to the fire combustion noise obtained in real time; that is, $\sum_{i=1}^{n}{|C_{i}|}^{2}$.

Depending on the trend line corresponding to the signal spectrum $C_{n}$, for the analog signal $x_{n}$ corresponding to the fire combustion noise obtained in real-time, the sum of the squares of the low frequency spectrum amplitude and the high frequency spectrum amplitude of each analog signal $x_{n}$ is calculated as $\sum_{i=1}^{n}{|C_{i}|}^{2}$. Finally, we write the evaluation value *Y* corresponding to the real-time fire combustion noise as follows:

(9) $$\begin{array}{c} Y=\frac{\sum_{i=1}^{n}{|C_{i}|}^{2}}{P_{f}}, \end{array}$$

where $C_{i}$ represents the spectral amplitude value of the analog signal of number *i* corresponding to the fire combustion noise obtained in real-time. We judge whether *Y* in Equation (9) is higher than the preset evaluation threshold. If Y is higher than the evaluation threshold, we determine that the fire combustion noise received in real-time represents the crown fire. Otherwise, it is judged that the real-time combustion noise represents the surface fire.

## 4. Analysis

### 4.1. Experimental Device

**Figure 7:**
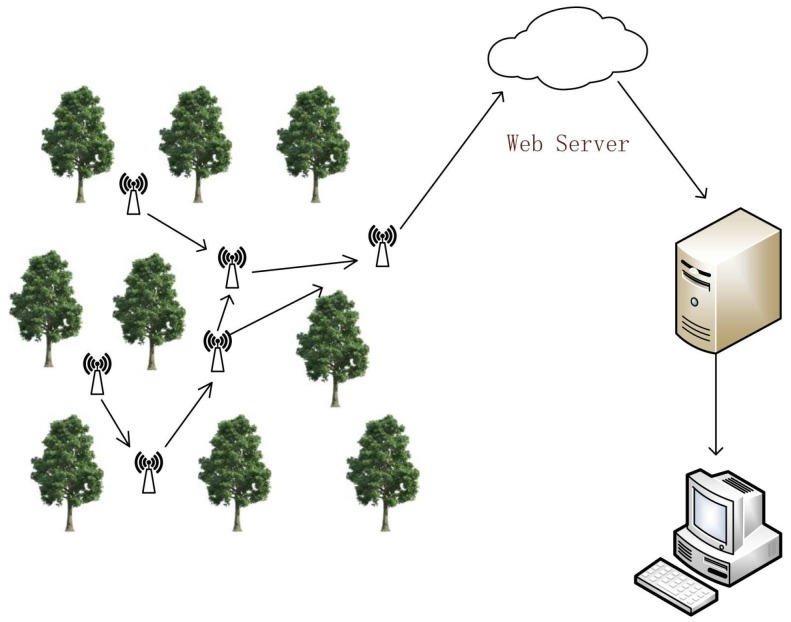
LoRa equipments deployed in forests.

To evaluate the performance of wildfire monitoring systems using sound spectrum analysis based on the Internet of Things, we randomly deployed a large number of sensor nodes and monitoring systems in the forest area. The data collected by sensors were forwarded to at least one sink node and ultimately to the remote terminal. Forest fire fighters monitor the area by analyzing these data by using sound spectrum analysis, as shown in Figure 7.

In our design, sensor nodes are equipped with rechargeable power devices that convert tree-energy into electricity energy. Each sensor is equipped with a tree-energy harvesting module, which means that the energy of the living trees can be converted into electricity; therefore, the energy equipment of all nodes can be recharged, and the transmission power of each sensor can be adjusted. Energy charging devices and energy storage devices, as energy replenishing elements, replace batteries used in traditional LoRa devices. The maximum communication distance per node is set to 3 km. Each node can communicate directly within its transmission range.

### 4.2. Tree-Energy Device Analysis

Photosynthesis and respiration are two essential life activities in the biological world for the carbon cycle. Plants perform photosynthesis during the day and release energy to the outside. At night, plants respire and store energy. Compared to the nighttime, the plant has less power inside the daytime; thus, the tree-energy device generates less electricity during the day than at night. Besides, photosynthesis and respiration of plants are susceptible to external influences, exceptionally water, light intensity and duration, and temperature.

**Figure 8:**
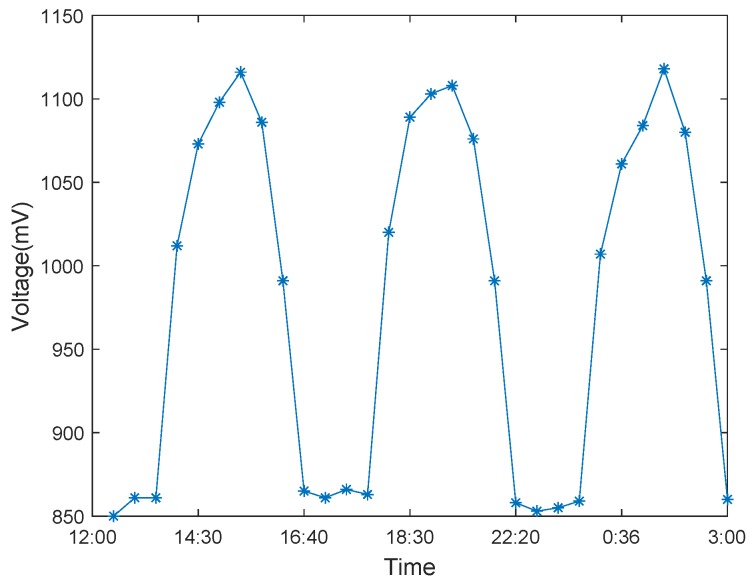
Tree-energy voltage variation in summer.

**Figure 9:**
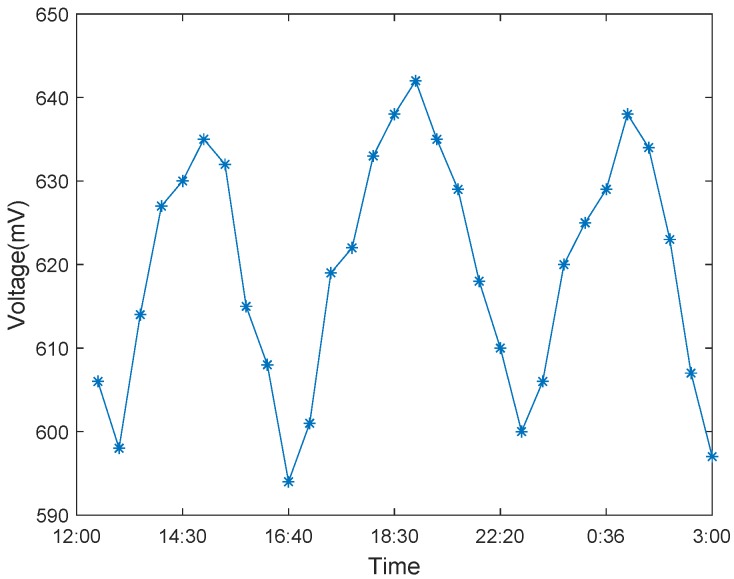
Tree-energy voltage variation in winter.

To measure the power generation performance of the tree-energy device, we utilized living trees such as metasequoia and eucalyptus as energy sources and analyzed the energy generated by them. We conducted a 3-day tree-energy voltage examination in summer and winter, respectively, as shown in Figure 8 and Figure 9. As expected, member nodes equipped with tree-energy devices can harvest bio-energy systematically throughout the day and quickly consume its energy supply. These records are consistent with circadian rhythms. The results show that light and water can easily influence the biological energy of living trees, and the change of energy is roughly sinusoidal in 24 h.

### 4.3. Results of Sound Spectrum Analysis

In the experiment, wildfire noise records collected by sound sensors were used for data analysis and the results of the analysis show that the sound spectral forms of surface fires and crown fires are different: the trend line amplitude of the ground fire noise spectrum gradually increases, and the trend line of the crown fire noise spectrum is a bell-shaped (Gaussian) trend line test. The research method in this paper is based on the Fourier transform of different analysis methods of the wildfire noise spectrum. Fast Fourier transform (FFT) means that the necessary noise power spectrum can be obtained from the recorded sound waveform. The purpose of this method is to identify the frequency response characteristics of noise under different wildfire types. The results are shown in the following figures.

**Figure 10:**
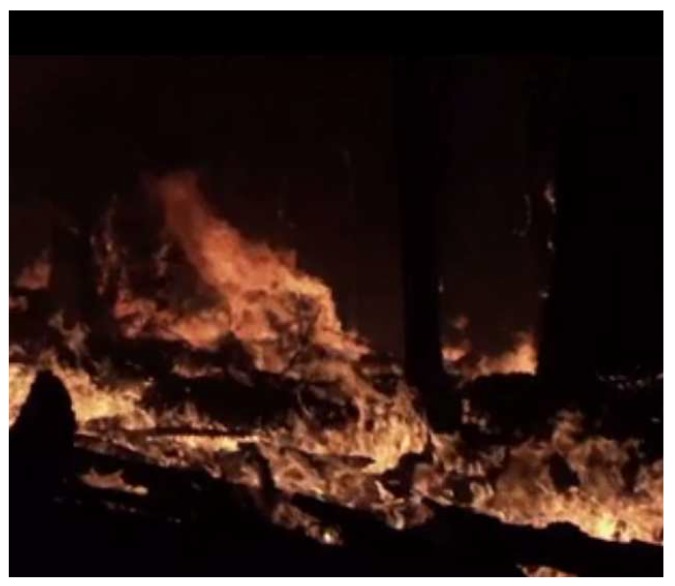
Initial crown fire.

**Figure 11:**
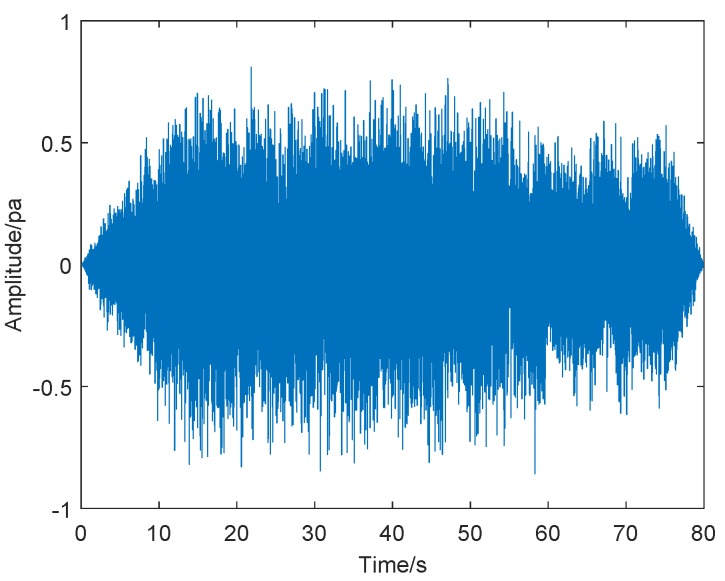
Time-domain sound signal.

**Figure 12:**
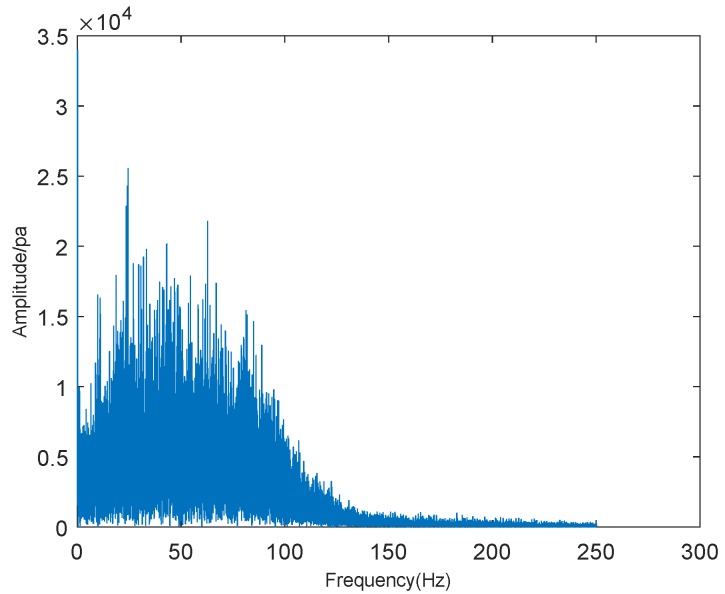
Frequency-domain sound signal.

Firstly, we took a screenshot of the initial crown fire captured by the surveillance video, as shown in Figure 10. The video shows the occurrence of a tree crown fire at night. In the video, we can see the flames floating around with the support of the wind, making the crown fire more serious and difficult to extinguish. We performed sound spectrum analysis of the 80 s, the audio of wildfire collected by sound sensors, and the results are shown in Figure 11 and Figure 12, where Figure 11 is the result of time-domain sound signal in the left channel, while Figure 12 represents frequency-domain sound signal of left channel.

Figure 11 illustrates that the sound signal in time domain is relatively stable and has no obvious changes. The audio signal is generally divided into two channels, the left channel and the right channel, to more accurately determine the exact location of different sound sources in the recording. However, when analyzing the sound spectrum with software, the monophonic channel is converted directly into stereo, and the waveform data of the left and right channels are consistent; hence, we do not need to analyze the time-domain sound signal and frequency-domain sound signal in the right channel.

Figure 12 shows that when the frequency of the crown fire sound reaches about 25 Hz, the waveform amplitude of sound spectrum analysis is the highest, which also indicates that the loudness of wildfire noise is the highest. In this figure, we also can estimate the frequency range of wildfire noise: 0–250 Hz. According to Equation (9), value *Y* corresponding to noise of wildfire combustion for Figure 10 is about 13.6, which is higher than the threshold value. Hence, it confirms the correctness of our proposed wildfire classification method.

**Figure 13:**
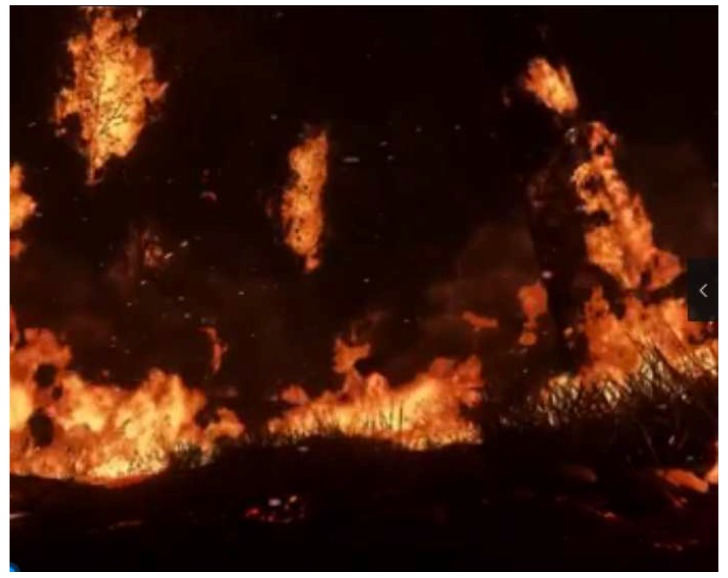
Strong crown fire.

**Figure 14:**
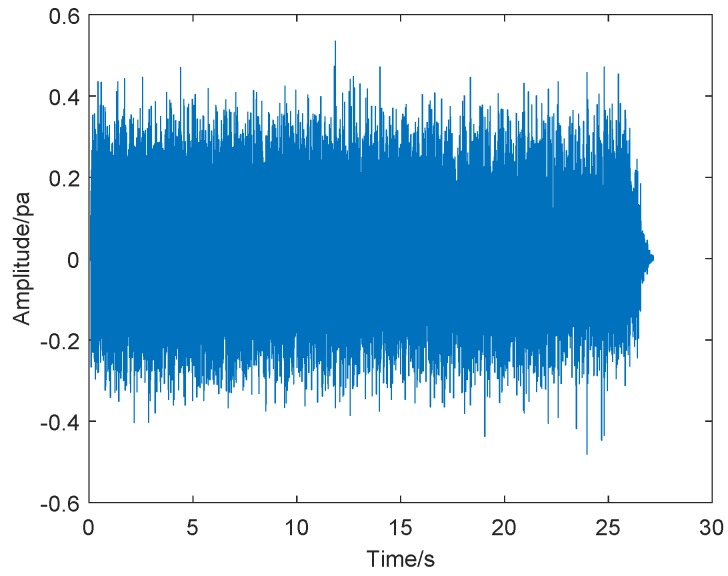
Time-domain sound signal.

**Figure 15:**
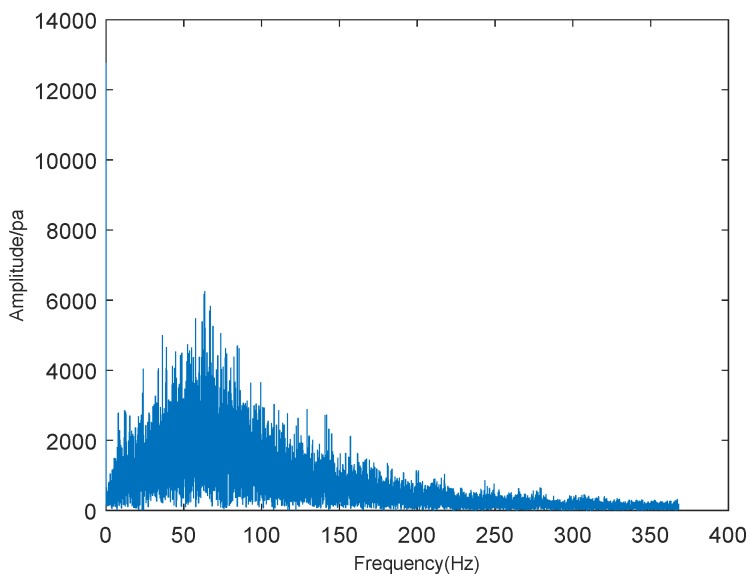
Frequency-domain sound signal.

Then, we analyzed a strong tree crown fire, the situation of the strong crown fire shown in Figure 13. Figure 14 and Figure 15 show the sound spectrum analysis results of 30s, the audio of wildfire’s sound spectrum analysis, which was attained by sound sensors, where Figure 14 is the result of time-domain sound signal in the left channel, while Figure 15 represents frequency-domain sound signal of left channel.

From Figure 14, we found that the sound signal in time domain was relatively stable and had no obvious changes. Figure 15 is sound frequency-amplitude waveform obtained by sound spectrum analysis. From this picture, we can see that when the sound frequency value of the strong crown fire is approximately 60 Hz, the amplitude is also at its maximum, which also manifests that wildfire noise is the loudest. In the figure, we also can see the frequency range of crown fire is relatively narrow and estimate its frequency range: 0–400 Hz.

Compared with Figure 12, Figure 15 describes that there is a little change in the frequency range of domain sound signals, ranging about from 0 to 350 Hz. In a word, we can define that the frequency range of crown fire is relatively narrow. By utilizing Equation (9), we calculated that the corresponding *Y* value of fire combustion noise for Figure 13 was about 18.3, which was higher than the threshold value. Therefore, it could more fully prove the effectiveness of the method we proposed in this paper.

To summarize, the trend line of noise spectrum acquired in the experiment gradually increases toward low frequency. The power spectrum trend line of the canopy fire noise has a distinct Gaussian type. The crown fire has a relatively narrow frequency range from 0 to 350 Hz.

**Figure 16:**
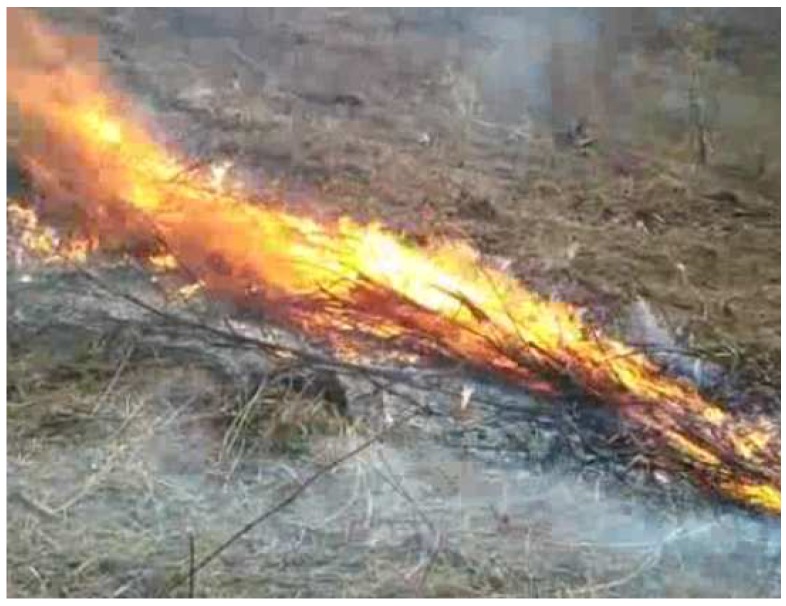
Surface fire.

**Figure 17:**
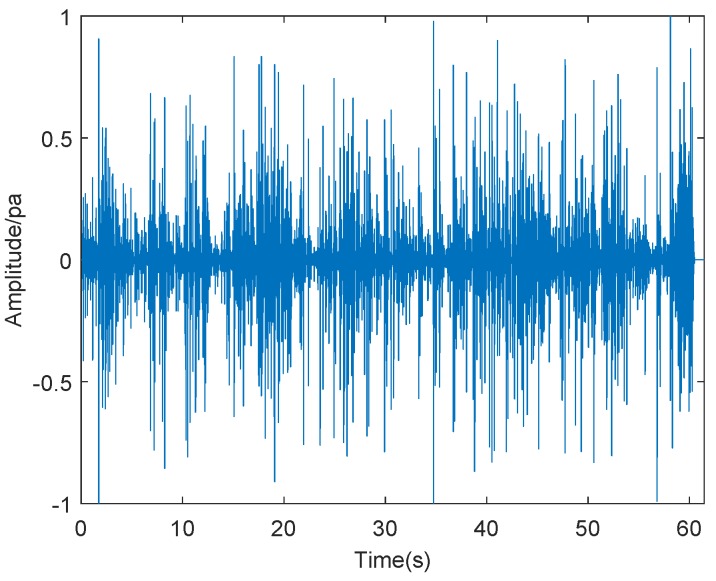
Time-domain sound signal.

**Figure 18:**
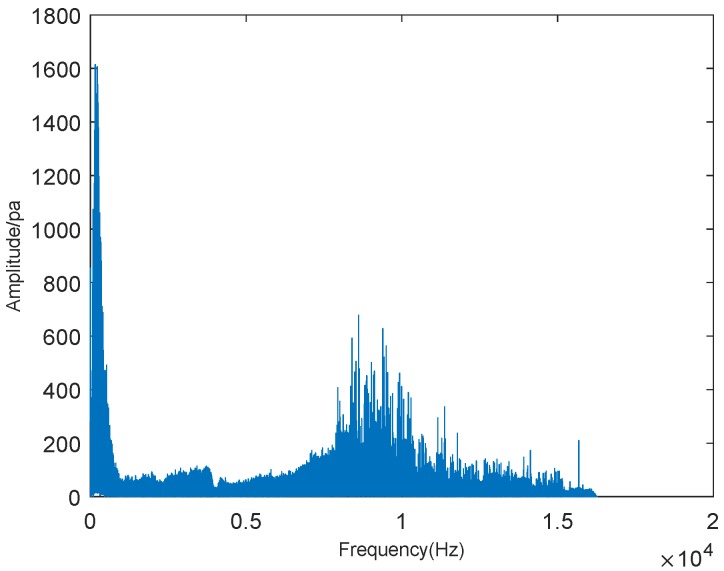
Frequency-domain sound signal.

Then, we performed sound spectrum analysis on the surface fire. The surface fire is similar to the picture depicted in Figure 16. The results of sound spectrum analysis on the surface fire are shown in the following figures. Figure 17 and Figure 18 show the results of sound spectrum analysis of wildfire for 60 s, which obtained by sound sensors, where Figure 17 is the result of time-domain sound signal in the left channel, while Figure 18 represents the frequency-domain sound signal of left channel.

As shown in Figure 17, it can be obviously observed that the time-domain sound signal fluctuates up and down and changes periodically. By compared with the time-domain sound signals of the crown fire, we can find that the time domain sound signal of surface fire is different from that of the crown fire. Therefore, we are sure that the waveform of the time-domain sound signals of different types of wildfire can be easily distinguished. Figure 18 plots that when the frequency of the surface fire sound reaches about 220 Hz, the waveform amplitude of sound spectrum analysis is the highest, which also manifests that wildfire noise is the loudest. In the figure, we also can estimate that the frequency range is from 0 to 15,000 Hz. Depending on Equation (9), we calculated the value *Y* of Figure 16 was about 5.1, which is lower than the threshold value. This also confirms the accuracy of our experimental method.

**Figure 19:**
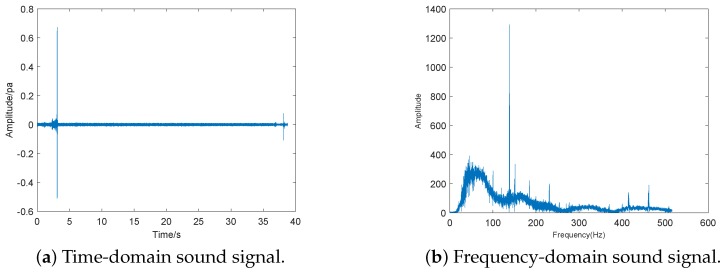
Sound spectrum of forest in the absence of wildfire.

Finally, in the absence of wildfire, we collected the sound of the forest and analyzed the sound spectrum. The results are shown in Figure 19, where the left figure is the result of the time-domain sound signal, while the right one represents the frequency-domain sound signal of the left channel. It is universally acknowledged that when there is no occurrence of wildfire, the sound of the forests we collect mostly comes from the birds and animals that inhabit the forest. While these sounds made by animals are relatively short, which is why the time-domain signal in figure (a) basically tends to zero, with few fluctuations. In figure b, we discover that both the frequency and amplitude value of sound made by animals is small relative to that of crown fire and surface fire.

### 4.4. Intuitive Comparison

**Figure 20:**
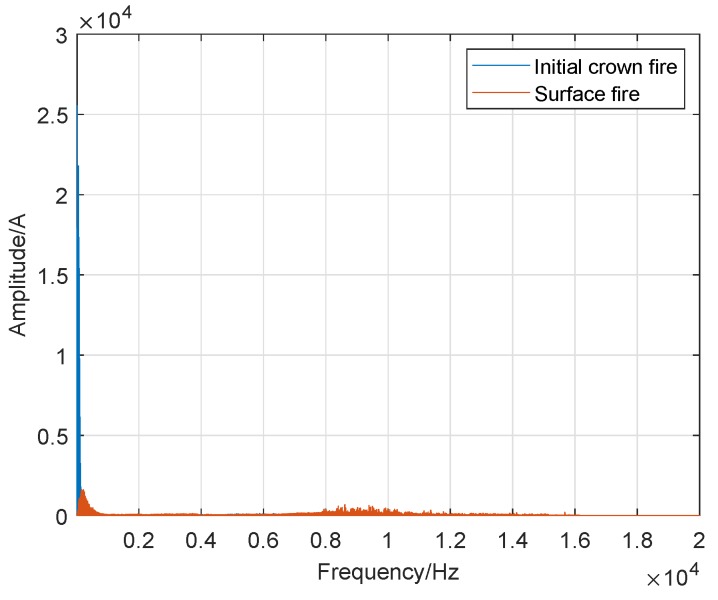
Comparison of initial crown fire and surface fire.

**Figure 21:**
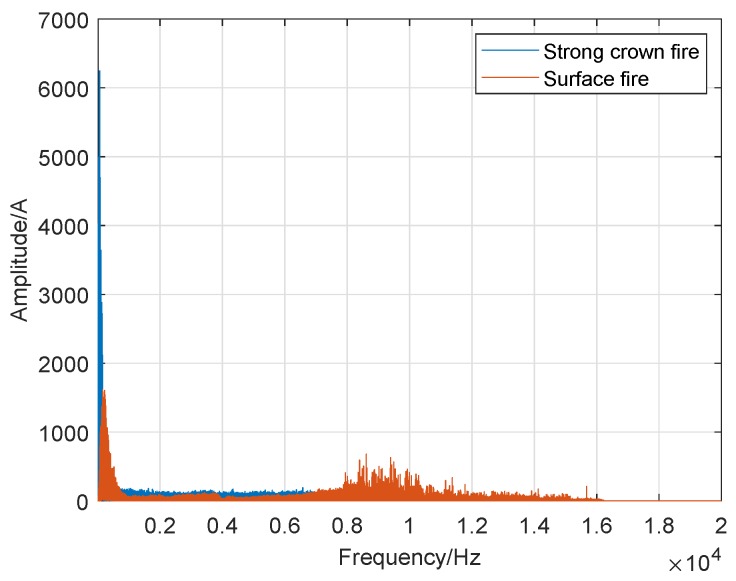
Comparison of strong crown fire and surface fire.

**Figure 22:**
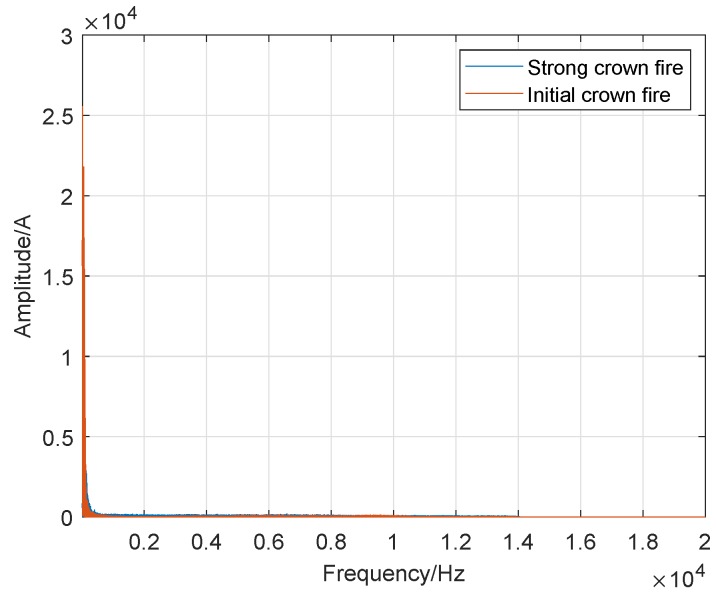
Comparison of strong crown fire and initial crown fire.

In the previous subsection, to accurately determine the sound frequency range of crown fire and surface fire, we used different frequency intervals to describe the variation between the frequency and amplitude of wildfire noise and display it in the corresponding diagram (spectrum plots). Therefore, we cannot make an intuitive comparison between them. To verify our statement, we used a consistent and wider frequency interval in all the corresponding spectrum plots: 0 Hz to 20 kHz in a logarithmic frequency scale. Besides, we put the spectrum of the crown fire and surface fire in the same picture as shown in Figure 20, Figure 21 and Figure 22.

The Figure 20 is the result of frequency-sound amplitude, which is obtained by comparing the acoustic spectrum analysis of the initial crown fire and the surface fire. To compare sound amplitude of the initial crown fire and the surface fire at the same frequency, the frequency range of the Abscissa of the image was set to 0–20,000 Hz. The figure plots that the sound frequency of the crown fire is very narrow, but its the value of sound amplitude is higher than that of surface fire, while surface fire is opposite to crown fire. The noise of surface fire has a frequency of up to 15,000 Hz, but its sound amplitude is relatively low and is almost invisible in the axis.

The Figure 21 is the result of frequency-sound amplitude from a comparison of the sound spectrum analysis of the strong crown fire and the surface fire. In Figure 21, we notice that on this axis, the sound frequency of the strong crown fire is narrower than that of the surface fire, while its sound amplitude value is higher than the surface fire’s sound amplitude, which is alike to Figure 20.

Distinguishing itself from Figure 20 and Figure 21, Figure 22 depicts the results of the acoustic spectrum analysis of the initial crown fire and the strong crown fire, and compares the results. In this image, in addition to the different sound amplitude, the waveforms of the other parts are coincident, which confirms that the sound spectrum analysis of crown fire has similar characteristics.

## 5. Conclusions

In this paper, a novel wildfire monitoring technology using sound spectrum analysis based on the Internet of Things is proposed. Some relevant research was conducted. First, according to the principle that different combustion materials produce different noises when burning, we analyzed the noise spectrum of wildfire and classified them for early fire detection. Second, it was found that because of the specific characteristics of plants, there is a constant potential voltage difference between the wooden parts of the plants and the surrounding soil, thereby providing a chance to improve the technology for collecting energy from the environment. Owing to that promising discovery, we designed an independent energy harvesting device, which can assemble the power from the living trees for maintaining the regular operation of sensor nodes. Third, for further the distance of traditional wireless communication, we employed the LoRa communication device, which transmits data collected by the sensor to the receiving end to implement remote connection. Finally, the collected sound data was analyzed by sound spectrum analysis and the results show that the frequency range of the crown fire is relatively small, ranging from 0 to 400 Hz, while the frequency range of the surface fire is about 0–15,000 Hz. To improve the accuracy of wildfire classification, we also designed a formal wildfire type determination and classification algorithm to judge wildfire types by calculating the value of evaluation value unY corresponding to fire noise depending on Equation (9). If *Y*, the evaluation value, is higher than the threshold, we can determine that the type of wildfire is crown fire. Otherwise, the kind of wildfire is surface fire.

## Figures and Tables

**Table 1:** Component symbols.

Component Symbol	Component Name
C_1_, C_2_	the first/second capacitor
S_1_, S_2_	the first/second switch
DC/DC	Direct current-Direct current converter
M_1_, M_2_	the first/second Micro controller Unit
